# Left Ventricular Remodeling in Hypertrophic Cardiomyopathy: An Overview of Current Knowledge

**DOI:** 10.3390/jcm10081547

**Published:** 2021-04-07

**Authors:** Beatrice Musumeci, Giacomo Tini, Domitilla Russo, Matteo Sclafani, Francesco Cava, Alessandro Tropea, Carmen Adduci, Francesca Palano, Pietro Francia, Camillo Autore

**Affiliations:** Cardiology, Department of Clinical and Molecular Medicine, Faculty of Medicine and Psychology, Sapienza University of Rome, 00189 Rome, Italy; beatrice.musumeci@uniroma1.it (B.M.); giacomo.comotini@gmail.com (G.T.); russodomitilla@gmail.com (D.R.); matteosclafani18@gmail.com (M.S.); francescocavauniroma1@gmail.com (F.C.); alessandro.tropea3@gmail.com (A.T.); carmenadduci@yahoo.it (C.A.); f.palano@gmail.com (F.P.); pietro.francia@gmail.com (P.F.)

**Keywords:** hypertrophic cardiomyopathy, left ventricular remodeling, end stage, risk stratification

## Abstract

While most patients with hypertrophic cardiomyopathy (HCM) show a relatively stable morphologic and clinical phenotype, in some others, progressive changes in the left ventricular (LV) wall thickness, cavity size, and function, defined, overall, as “LV remodeling”, may occur. The interplay of multiple pathophysiologic mechanisms, from genetic background to myocardial ischemia and fibrosis, is implicated in this process. Different patterns of LV remodeling have been recognized and are associated with a specific impact on the clinical course and management of the disease. These findings underline the need for and the importance of serial multimodal clinical and instrumental evaluations to identify and further characterize the LV remodeling phenomenon. A more complete definition of the stages of the disease may present a chance to improve the management of HCM patients.

## 1. Introduction

Left ventricular (LV) remodeling consists of changes in the ventricular architecture, such as dilation of the cavity and wall thickening or thinning, due to a combination of pathologic myocyte hypertrophy, myocyte apoptosis, myofibroblast proliferation, and interstitial fibrosis [1]. Although originally described after myocardial infarction [2], LV remodeling can occur in physiological conditions, i.e., athlete’s heart [3], as well as in several forms of myocardial injury and cardiac disease.

In recent years, it has been found that remodeling of the LV morphology occurs in patients with hypertrophic cardiomyopathy (HCM) during their life course [4]. LV remodeling in HCM has an important pathophysiologic impact, significantly affecting the natural history and management of this disease [4,5].

In the present work, we will review the features of LV remodeling in HCM and its influence on the natural history of the disease. 

## 2. Natural History of HCM

The clinical course of HCM is extremely heterogeneous, resulting from a complex interaction of LV hypertrophy, LV remodeling, and several functional alterations, including diastolic impairment, myocardial ischemia, outflow tract obstruction, and arrhythmias [6]. Because the severity of each of these morphologic and functional abnormalities varies greatly among individuals, the clinical spectrum of HCM differs from asymptomatic to dramatic phenotypic expression such as sudden death [7]. 

Over time, the common perception and natural history of HCM have significantly changed. In its first description, HCM was considered to be a disease with an unfavorable prognosis and an annual mortality between 4% and 6% [8]. However, at that time, most of the information about the clinical course and prognosis of HCM came from only two referral centers. In the following years, with the introduction of new diagnostic methods and the publication of findings from non-selected cohorts from several other centers, our understanding of HCM improved [9,10,11]. Today, it is clearly recognized that HCM can be diagnosed in any phase of life and its annual mortality rate is approximately 1.4% [7]. When a diagnosis is made in young individuals (<25 years), the disease may be less favorable and the annual mortality rate is usually higher, with sudden cardiac death as the main cause [6,12,13]. However, when the disease is diagnosed in adults, it is often characterized by a stable course, with effective symptom control by medical therapy and a normal life expectancy [14]. Some HCM subgroups are characterized by a worse prognosis: (1) patients with a high risk of sudden death, with no symptoms of heart failure; (2) those with progressive symptoms of heart failure and, hence, worsening of quality of life with or without LV obstruction and in the absence of systolic dysfunction; (3) a subset of patients developing so-called end-stage disease, characterized by LV systolic dysfunction or apical aneurysms. Furthermore, the development of atrial fibrillation, mostly secondary to diastolic dysfunction and high LV filling pressure, has an unfavorable hemodynamic impact on the clinical course of HCM and is associated with a significant risk of thromboembolism and stroke [15]. 

A variety of different pharmacologic and non-pharmacologic management strategies have been developed over the last 15 years, including implantable defibrillators, advances in surgical myectomy, alcohol ablation, or heart transplant, altering the natural history and disease course for many HCM patients [16]. In particular, the increased understanding of sudden cardiac death risk stratification strategies has enabled more reliable selection of those HCM patients likely to achieve primary prevention of sudden death with an implantable defibrillator. Consequently, heart failure has become the predominant cause of morbidity and mortality in patients with HCM, responsible for as many as 60% of HCM-related deaths [16,17].

## 3. Pathophysiological Mechanisms of LV Remodeling in HCM

**Figure 1:**
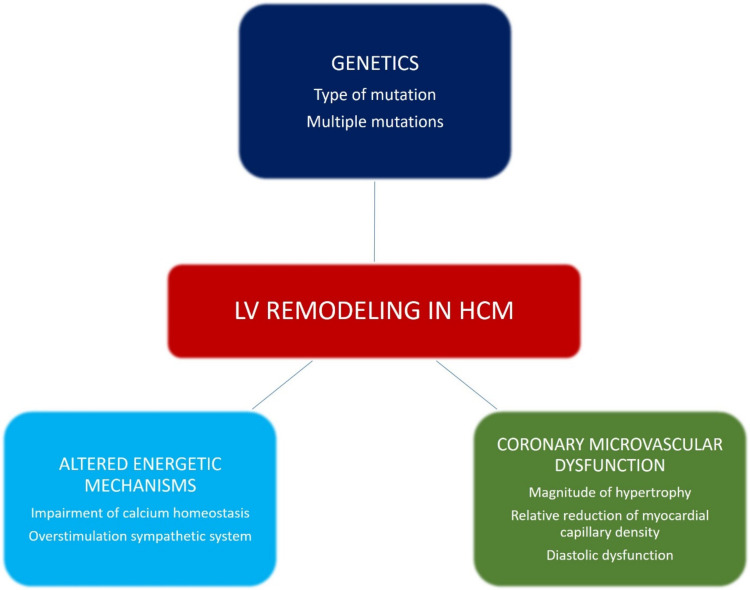
Pathophysiological mechanisms involved in left ventricular (LV) remodeling in hypertrophic cardiomyopathy (HCM).

The interaction of multiple mechanisms is thought to be responsible for LV remodeling in HCM. While a complex genetic interplay is implicated mainly in the growth and magnitude of LV hypertrophy, other specific myocardial and vascular mechanisms (mainly causing ischemia and fibrosis) occur during the natural course of HCM and may result in progressive LV thinning, with or without systolic dysfunction. The pathophysiological mechanisms involved in the process of LV remodeling in HCM are summarized in Figure 1.

### 3.1. Genetics

HCM is a sarcomeric disease determined from mutations in a number of genes encoding cardiac contractile and Z-disk proteins [18]. Mutations in sarcomeric genes generally cause the disease through single amino acid substitutions in proteins that become incorporated into the sarcomere and act as poison peptides with an impairment of normal sarcomere function [19]. Contrastingly, most myosin-binding protein C (MYBPC3) mutations result in insufficient protein production for normal sarcomere function (haploinsufficiency) [20]. HCM-causing mutations may exert various adverse effects on cardiomyocyte intracellular calcium and energy handling, accounting for early diastolic abnormalities in genotype-positive patients, even before the development of LV hypertrophy [21]. Moreover, there is a significant HCM phenotypic variability, in which regulatory genes and other factors are likely implicated, including environmental modifiers [22]. Finally, up to 5% of families carry two distinct disease-causing gene mutations. Multiple gene mutations occurring in HCM families typically result in a more severe clinical phenotype with an earlier onset of disease and a higher incidence of sudden cardiac death or heart failure events [19].

### 3.2. Altered Energetic Mechanisms

HCM-causing mutations compromise cardiomyocyte energetic balance, generally enhancing calcium sensitivity, maximal force production, and ATPase activity. The consequent chronic impairment of cardiomyocyte calcium homeostasis represents a major common pathway to the anatomic (hypertrophy, myofiber disarray, and fibrosis) and functional features (diastolic dysfunction) characteristic of HCM [23]. Calcium-regulated signaling pathways are activated with several downstream effects, such as cardiac remodeling and, possibly, apoptosis. Moreover, the increased contractile status causes persistent stimulation of the sympathetic system, which, in turn, may contribute to regulatehypertrophy [24].

### 3.3. Coronary Microvascular Dysfunction

Coronary microvascular dysfunction, subtended by structurally remodeled small coronary vessels, is an important feature of HCM, associated with LV remodeling and heart failure. It appears to be genetically driven and relatively independent of hypertrophy [25]. In HCM, different mechanisms contribute to myocardial ischemia separately or simultaneously in the same patient. First of all, myocardial oxygen requirement is increased due to the magnitude of hypertrophy, whereas the relative myocardial capillary density may be reduced, thus lowering the ischemic threshold [26]. Furthermore, coronary vascular resistance may be enhanced due to systolic compressive deformation, LV outflow tract obstruction, and higher diastolic wall tension secondary to impaired LV relaxation and filling [27,28]. All of these factors lead to progressive myocardial cell death and replacement fibrosis, which is hypothesized to play an essential role in the pathogenesis of LV wall thinning [29]. 

Accurate quantitative assessment of microvascular dysfunction and myocardial ischemia may be performed by using an electrocardiogram, echocardiography, or myocardial scintigraphy, but examination of the vasodilator response to dipyridamole in positron emission tomography is considered the method of choice for the assessment of maximal regional and global flow [29]. Furthermore, cardiac magnetic resonance provides further information with late gadolinium enhancement (LGE), which may show areas where replacement fibrosis has occurred following microvascular ischemia and focal necrosis [30]. 

## 4. Types of LV Remodeling in HCM

**Figure 2:**
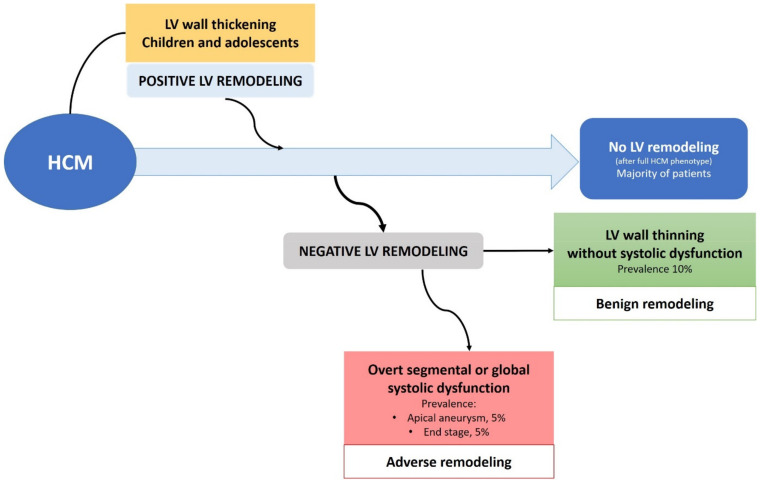
Different patterns of LV remodeling occurring during the clinical course of HCM.

LV remodeling occurs spontaneously among patients with HCM in several ways: (1) wall thickening, otherwise defined as positive remodeling; (2) wall thinning without impairment of LV systolic function, defined as benign remodeling; (3) a gradual wall thinning process resulting in the loss of LV systolic function or the development of LV aneurysms, defined as adverse remodeling (Figure 2).

### 4.1. Positive Remodeling

The phenotypic expression of HCM usually develops during adolescence [5]. Studies on genotype positive–phenotype negative (i.e., non-hypertrophic) HCM individuals have shown that hypertrophy presents at a younger age in the majority of cases, although it has rarely been reported to occur as late as in the sixth decade of life [31,32,33]. Once the HCM phenotype is fully expressed, the morphologic course of the disease is typically stable. Hence, progression of LV hypertrophy with thickening of the hypertrophied myocardium (i.e., positive LV remodeling) has been reported to be mostly limited to children or young HCM individuals in the early stages of the disease [4].

In a seminal study investigating 39 children aged < 15 years with a diagnosis of HCM or family history of the disease, Maron and colleagues first comprehensively described the process of positive remodeling [34]. In 22 out of the 39 patients, including five with an initially negative phenotype, a marked progression of hypertrophy occurred, with an average increase of 12 mm. In all cases, hypertrophy progression was limited to adolescence, up to 19 years of age [34]. The increase in wall thickness was associated with only mild increases in cavity dimension.

Subsequently, the same group investigated the occurrence of positive remodeling in 65 adult HCM patients (20 to 50 years of age), with at least 3 years of follow-up [35]. None of the study patients showed an increase of ≥5 mm in any of the LV segments. Based on these results, positive remodeling was believed to be related to body growth and maturation [4].

However, in 1985, Domenicucci and colleagues presented results from a cohort of 39 adult HCM patients, among whom four showed mild positive remodeling (the four patients being 17, 49, 49, and 53 years of age) [36]. More recently, Doolan and colleagues have reported findings from serial echocardiographic evaluations in a cohort of 62 HCM patients [37]. Positive remodeling was observed in both younger (≤30 years of age) and older (>30 years) patients. However, the mean increase in LV wall thickness was 6 mm versus 1.7 mm, respectively. Moreover, the study showed that the presence of the angiotensin-converting enzyme (ACE) gene deletion polymorphism (D/D) was associated with positive remodeling, as compared to other ACE polymorphisms, and was independent of age, body mass, and resting blood pressure. Hence, it is likely that other mechanisms beyond physiological body growth contribute to positive remodeling in HCM. This is in line with a previous investigation from the same Australian group that found marked positive remodeling in HCM patients aged 10 to 20 years (from a mean of 15.9 mm to 19.2 mm in the interventricular septum), but also a very small increase in patients of 21–40 years of age (from 16.0 mm to 17.8 mm) [38].

Importantly, positive remodeling is seldom associated with clinical manifestation or symptom progression.

More recent longitudinal studies are lacking; nevertheless, positive remodeling differing from that described in past analyses (mostly relying on echocardiography) has not been reported to date. Thus, it appears to be a process occurring almost exclusively in adolescence. Although diagnosis has been shown to occur at an older age in contemporary HCM cohorts [39], the demonstration of positive LV remodeling after diagnosis has not yet been reported. 

**Figure 3:**
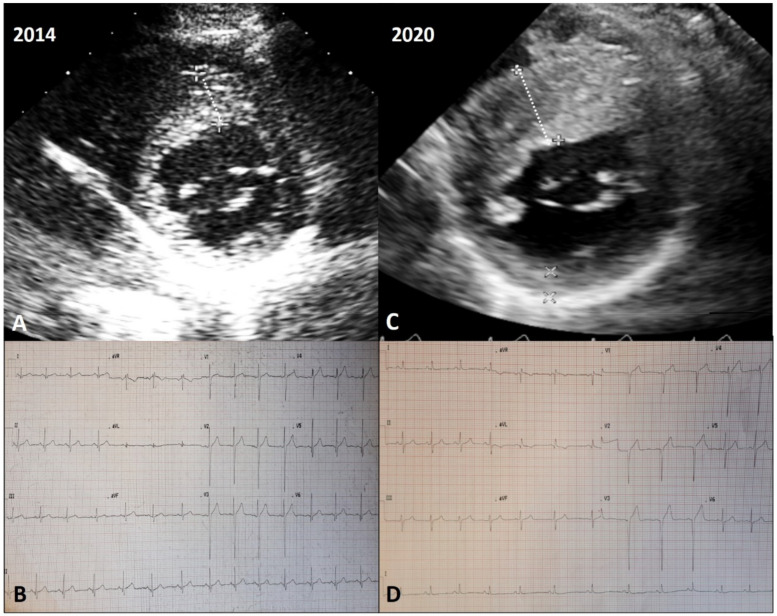
Echocardiographic and electrocardiographic findings in a patient with positive LV remodeling. A young male was diagnosed with non-obstructive HCM during a familial screening (mother affected) at 14 years of age, and since then followed at our outpatient clinic. At initial evaluation, the echocardiogram showed a maximal wall thickness of 16 mm at the level of the anterior septum (**A**), with signs of LV hypertrophy in the electrocardiogram (**B**). After 6 years, a significant increase in maximal wall thickening (24 mm) was found via echocardiography (**C**). The electrocardiogram (ECG) showed left axis deviation and the occurrence of Q waves in V1–V3 (**D**). At cardiac magnetic resonance imaging, the presence of intramural late gadolinium enhancement at the level of maximal hypertrophy was detected, and 24-h ECG Holter monitoring revealed a run of non-sustained ventricular tachycardia of 8 beats at rate of 160/min. He was implanted with a subcutaneous implantable cardioverter defibrillator for primary prevention.

An explicative case of positive remodeling is reported in Figure 3.

### 4.2. Benign Remodeling

Since the 1980s, it has been observed that younger HCM patients show substantially more severe and diffuse LV hypertrophy than older patients do [40,41,42]. At that time, the phenomenon was hypothesized to be due to both a higher incidence of sudden death in subjects with severe hypertrophy and a progressive reduction over time in the wall thickness. After the year 2000, by means of serial echocardiographic observations, it was confirmed that the reduction in myocardial thickness contributes substantially to the lower prevalence of severe LV hypertrophy in adults [43]. Therefore, it was recognized that a process of LV remodeling occurs during the natural course of HCM, with a combination of several structural and functional modifications, including a mild reduction in LV systolic function (i.e., LV ejection fraction in the low–normal range) [44], moderate to severe diastolic dysfunction [45], remodeling and dilation of the left atrium and consequent onset of atrial fibrillation [15], thinning of the LV walls up to normal values [4], spontaneous reduction in or loss of LV outflow obstruction [46], and advanced microvascular dysfunction [47]. However, most of these modifications, affecting only 15–20% of patients, did not cause hemodynamic imbalance and overt LV systolic dysfunction [5], and their prognostic implications were not clarified. Recently, a process of LV remodeling characterized by gradual LV wall thinning without systolic dysfunction and with a benign clinical course has been demonstrated to occur in around 10% of patients with HCM over the long term [48]. This benign remodeling pattern is characterized by a gradual process of wall thinning with minor changes of cavity dimension and systolic function, which both remain within the normal limits. Patients with benign remodeling present a benign outcome over a long-term period, with HCM-related morbidity and mortality similar to patients without LV remodeling and distinct from patients with end-stage HCM and apical aneurysm (i.e., adverse remodeling) [48]. The reason why some patients develop overt LV dysfunction while others present benign remodeling is still unknown, but it is reasonable to believe that other factors beyond time and magnitude of LV hypertrophy—including genotype and therapy—may cooperate in determining this process. 

**Figure 4:**
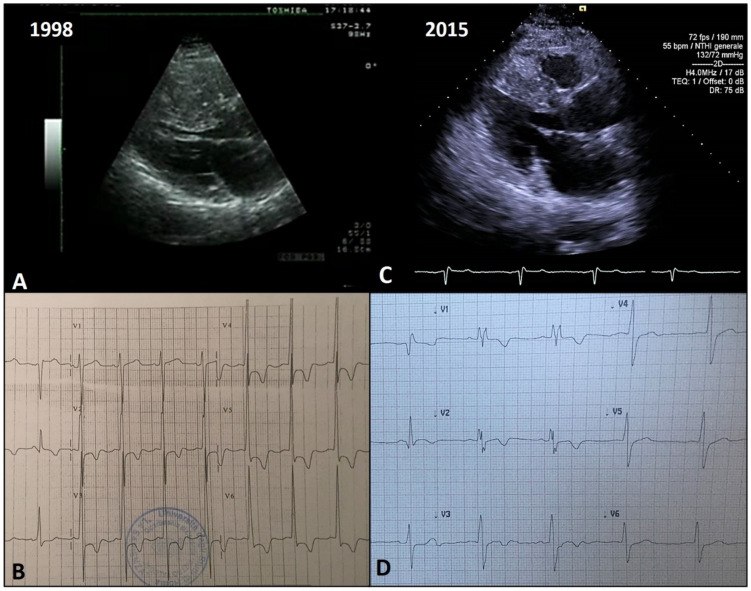
Echocardiographic and electrocardiographic findings in a patient with benign LV remodeling. A 49-year-old male patient received a diagnosis of non-obstructive HCM, with evidence of massive LV hypertrophy (maximal wall thickness of 37 mm at the level of the anterior septum (**A**)) in the echocardiogram. The electrocardiogram showed signs of LV hypertrophy and inverted T wave in precordial leads (**B**). At that time, the patient was implanted with a cardioverter defibrillator for primary prevention. After 17 years, the echocardiogram showed a progressive, significant reduction in maximal LV wall thickness, up to 24 mm at the last evaluation (**C**). Concurrently, a reduction in QRS amplitude and an increase in QRS duration were noted in the electrocardiogram (**D**). His clinical conditions are stable and he has never experienced any implantable cardioverter defibrillator shocks.

An explicative case of benign remodeling is reported in Figure 4.

### 4.3. Adverse Remodeling

**Figure 5:**
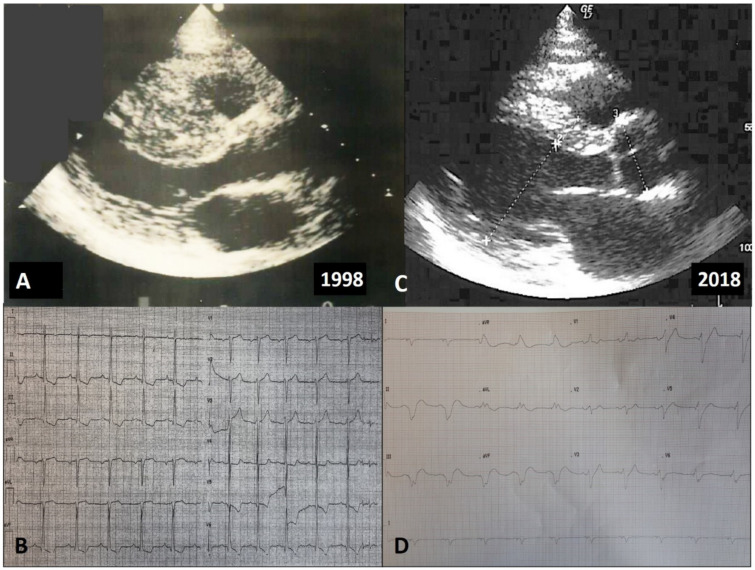
Echocardiographic and electrocardiographic findings in a patient with adverse LV remodeling, characterized by LV wall thinning and loss of systolic function. This is the case of a 38-year-old male with familial HCM. At first evaluation, the echocardiogram showed a maximal wall thickness of 27 mm at the level of the anterior septum (**A**), and the electrocardiogram showed signs of LV hypertrophy (**B**). After 20 years, he had an episode of decompensated heart failure. At admission in our cardiology ward, the echocardiogram showed a reduction in maximal wall thickness, which was 14 mm at the level of the anterior septum, and an enlargement of the LV cavity, with impaired systolic function (LV ejection fraction 20% (**C**)). The electrocardiogram showed sinus rhythm, first-degree atrioventricular block, extreme left axis deviation, right bundle branch block, and reduction in voltages (**D**). He underwent implantation of a biventricular cardioverter defibrillator. Coronary angiogram was normal. After 1 year, the patient died while enlisted for heart transplantation.

Adverse remodeling in HCM patients is defined by the presence of unfavorable LV structural modifications, usually associated with clinical deterioration, eventually determining the so-called end-stage phase of the disease [4,5,48]. When the myocardial replacement scarring process is particularly severe, the morpho-functional manifestations of adverse remodeling span between two extremes: a “hypokinetic-dilated” form, characterized by spherical remodeling of the LV, regression of hypertrophy up to severe systolic pump failure, enlarged ventricular chambers, and increased end-diastolic and end-systolic volumes (Figure 5) [49,50], and a “restrictive” form, characterized by a small and stiff LV with extreme diastolic dysfunction and only mildly or moderately impaired systolic function [51]. Notably, only the first pattern of advanced disease progression has been historically defined as the end-stage phase of HCM, with systolic dysfunction (LV ejection fraction <50%) required for defining the condition. Nevertheless, the latter pattern is increasingly recognized, and HCM patients with the restrictive evolution are considered, nowadays, to have an equally unfavorable adverse remodeling, resulting in a different “phenotype” of end-stage [51].

Moreover, a particular pattern of localized adverse remodeling is the LV apical aneurysm, defined as a discrete thin-walled dyskinetic or akinetic segment of the most distal portion of the LV chamber, associated with transmural scarring in the absence of obstructive atherosclerotic coronary artery disease [52,53]. Apical aneurysms occur most commonly in patients with two distinctive LV morphological forms. The first is an “hourglass” shape, with mid-ventricular maximal wall thickness and considerably less or no hypertrophy in the distal and proximal portions of the LV. In these patients, intraventricular pressure gradients are often present due to the mid-systolic contact of the septum and the LV free wall. In the second pattern, so-called “apical HCM” hypertrophy is predominant in the distal portion of the LV [52,53]. The dyskinetic/akinetic apical aneurysm may provide a structural basis for ventricular arrhythmias and intracavitary thrombus formation [52,53]. 

**Figure 6:**
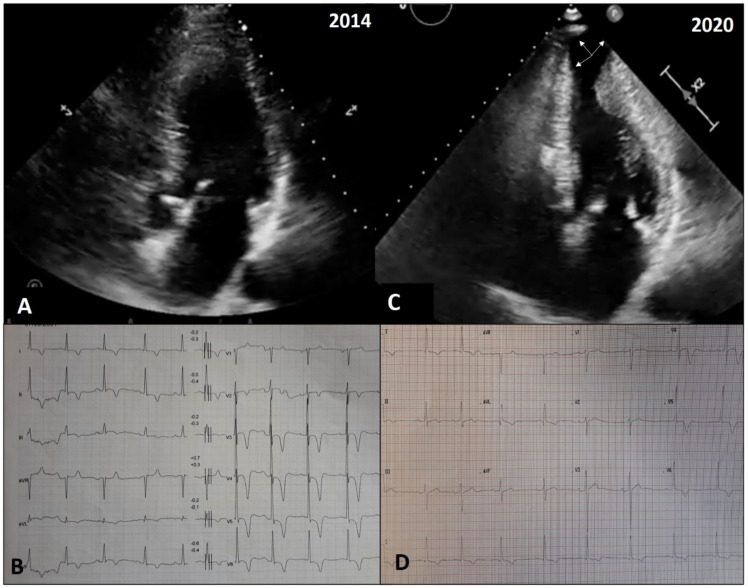
Echocardiographic and electrocardiographic findings in a patient with adverse LV remodeling, characterized by LV wall thinning and development of apical aneurysm. A 66-year-old male with familial HCM was diagnosed and evaluated at our outpatient clinic for the first time in 2014. Apical hypertrophy was evident in the echocardiogram (**A**), with signs of LV hypertrophy and deep inverted T wave in precordial leads in the electrocardiogram (**B**). After 6 years, we observed a significant remodeling of the LV apex with the development of a large aneurysm (arrows, **C**). As the apical aneurysm developed, QRS complex amplitude decreased and negative T waves became positive in V1–V3 and biphasic in V4 (**D**). The patient underwent implantation of a cardioverter defibrillator for primary prevention.

Close clinical surveillance with careful serial electrocardiographic and echocardiographic evaluation, cardiac magnetic resonance imaging, cardiopulmonary testing, and biomarkers can be valuable for early identification of the subgroup of patients in which adverse remodeling is occurring [54]. For example, serial electrocardiograms recorded before and after the development of LV apical aneurysms showed a notable progressive increase in the QRS complex duration and fragmentation along with a decrease in the QRS complex amplitude, a gradual and persistent ST segment elevation with depth reduction of negative T waves, and positivization of negative T waves in V3–V6 (Figure 6) [55]. Moreover, the information provided by contrast cardiac magnetic resonance imaging is crucial, since it is able to quantify and localize the deposition of fibrous tissue through LGE [43,56,57]. An LGE progression of ≥1.5% g/year in serial cardiac magnetic resonances was found to be indicative of adverse remodeling in terms of end-stage evolution and apical aneurysm occurrence [56].

The outcome of HCM patients with adverse remodeling is poor [4,58,59]. In previous end-stage cohorts, mortality was up to 11%/year [49]. In a recent study of contemporary end-stage HCM patients, mortality was lower, at about 1.9%/year [58], but still exceeding that of HCM patients without end-stage disease by 10-fold (0.2%/year; *p* < 0.001). The rate of progression from NYHA functional class I/II to advanced heart failure was 5.2%/year. Finally, the arrhythmic risk of end-stage patients remained high with a 2.4%/year incidence rate of appropriate implantable cardioverter defibrillator interventions [58]. The substantial improvement in mortality among end-stage HCM patients is most likely attributable to a number of contemporary management strategies, such as primary prevention implantable cardioverter defibrillators or selection for heart transplantation and LV assist devices [58]. Indeed, approximately 20% of patients with LV aneurysms undergo potentially life-saving implantable cardioverter defibrillator interventions, terminating ventricular tachyarrhythmias, with a risk 5-fold greater than that of HCM patients without aneurysms [52,53].

Two explicative cases of adverse remodeling are reported in Figure 5 and Figure 6.

## 5. Conclusions

The LV remodeling process has an important impact on the phenotypic expression, clinical course, and management of patients with HCM. Positive remodeling, identified by the progressive thickening of the hypertrophied myocardium, has generally been related to the growth and full expression of the disease. On the contrary, over the long-term course of the disease, gradual LV wall thinning without systolic dysfunction (benign remodeling) or severe, adverse LV remodeling may develop. Benign remodeling has been demonstrated to occur without any important impact on outcome as compared to patients without LV remodeling. Adverse remodeling, characterized by extensive fibrotic replacement and loss of LV systolic function, severe diastolic impairment, or the occurrence of an LV aneurysm, is associated with a very poor prognosis. These aspects of HCM natural history underline the need for and the importance of serial clinical, electrocardiographic, and echocardiographic evaluations to identify and further characterize the LV remodeling phenomenon over time. A comprehensive definition of the stages of the disease may present a chance to improve the management of HCM patients.

## References

[1] Eaton L.W., Weiss J.L., Bulkley B.H., Garrison J.B., Weisfeldt M.L. (1979). Regional cardiac dilatation after acute myocar- dial infarction: Recognition by two- dimensional echocardiography. N. Engl. J. Med..

[2] McKay R.G., Pfeffer M.A., Pasternak R.C., Markis J.E., Come P.C., Nakao S., Alderman J.D., Ferguson J.J., Safian R.D., Grossman W. (1986). Left ventricular remodeling after myocardial infarction: A corollary to infarct expansion. Circulation.

[3] Huston T.P., Puffer J.C., Rodney W.M. (1985). The athletic heart syndrome. N. Engl. J. Med..

[4] Maron B.J., Spirito P. (1998). Implications of Left Ventricular Remodeling in Hypertrophic Cardiomyopathy. Am. J. Cardiol..

[5] Olivotto I., Cecchi F., Poggesi C., Yacoub M.H. (2012). Patterns of disease progression in hypertrophic cardiomyopathy: An individualized approach to clinical staging. Circ. Heart Failure.

[6] Elliott P.M., Anastasakis A., Borger M.A., Borggrefe M., Cecchi F., Charron P., Hagege A.A., Lafont A., Limongelli G., Mahrholdt H. (2014). ESC Guidelines on diagnosis and management of hypertrophic cardiomyopathy. The task force for the diagnosis and management of hypertrophic cardiomyopathy of the European Society of Cardiology (ESC). Eur. Heart J..

[7] Maron B.J. (2018). Clinical course and management of hypertrophic cardiomyopathy. N. Engl. J. Med..

[8] Swan D.A., Bell B., Oakley C.M., Goodwin J. (1971). Analysis of symptomatic course and prognosis and treatment of hypertrophic obstructive cardiomyopathy. Br. Heart J..

[9] Spirito P., Chiarella F., Carratino L., Berisso M.Z., Bellotti P., Vecchio C. (1989). Clinical course and prognosis of hypertrophic cardiomyopathy in an outpatient population. N. Engl. J. Med..

[10] Maron B.J., Spirito P. (1993). Impact of patient selection biases on the perception of hypertrophic cardiomyopathy and its natural history. Am. J. Cardiol..

[11] Cecchi F., Olivotto I., Montereggi A., Santoro G., Dolara A., Maron B.J. (1995). Hypertrophic cardiomyopathy in Tuscany: Clinical course and outcome in an unselected regional population. J. Am. Coll. Cardiol..

[12] Spirito P., Autore C., Rapezzi C., Bernabò P., Badagliacca R., Maron M.S., Bongioanni S., Coccolo F., Estes N.M., Barillà C.S. (2009). Syncope and Risk of Sudden Death in Hypertrophic Cardiomyopathy. Circulation.

[13] Begley D.A., Mohiddin S.A., Tripodi D., Winkler J.B., Fananapazir L. (2003). Efficacy of implantable cardioverter-defibrillator for primary and secondary prevention of sudden cardiac death in hypertrophic cardiomyopathy. PACE.

[14] Ho C.Y., Day S.M., Ashley E.A., Michels M., Pereira A.C., Jacoby D., Cirino A.L., Fox J.C., Lakdawala N.K., Ware J.S. (2018). Genotype and Lifetime Burden of Disease in Hypertrophic Cardiomyopathy. Circulation.

[15] Olivotto I., Cecchi F., Casey S.A., Dolara A., Traverse J.H., Maron B.J. (2001). Impact of atrial fibrillation on the clinical course of hypertrophic cardiomyopathy. Circulation.

[16] Autore C., Musumeci M.B. (2020). The natural history of hypertrophic cardiomyopathy. Eur. Heart J. Suppl..

[17] Maron B.J., Rowin E.J., Casey S.A., Maron M.S. (2016). How hypertrophic cardiomyopathy became a contemporary tratabile genetic disease with low mortality. Shaped by 50 years of clinical research and practice. JAMA Cardiol..

[18] Watkins H., Ashrafian H., Redwood C. (2011). Inherited Cardiomyopathies. N. Engl. J. Med..

[19] Kelly M., Semsarian C. (2009). Multiple mutations in genetic cardiovascular disease: A marker of disease severity. Circ. Cardiovasc. Genet..

[20] Van Dijk S.J., Dooijes D., dos Remedios C., Michels M., Lamers J.M.J., Winegrad S., Schlossarek S., Carrier L., ten Cate F.J., Stienen G.J.M. (2009). Cardiac myosin-binding protein C mutations and hypertrophic cardiomyopathy: Haploinsufficiency, deranged phosphorylation, and cardiomyocyte dysfunction. Circulation.

[21] Ho C.Y. (2009). Hypertrophic cardiomyopathy: Preclinical and early phenotype. J. Cardiovasc. Trans. Res..

[22] Marian A.J., Roberts R. (2001). The molecular genetic basis for hypertrophic cardiomyopathy. J. Mol. Cell. Cardiol..

[23] Ashrafian H., McKenna W.J., Watkins H. (2011). Disease pathways and novel therapeutic targets in hypertrophic cardiomyopathy. Circ. Res..

[24] Schäfers M., Dutka D., Rhodes C.G., Lammertsma A.A., Hermansen F., Schober O., Camici P.G. (1998). Myocardial presynaptic and postsynaptic autonomic dysfunction in hypertrophic cardiomyopathy. Circ. Res..

[25] Olivotto I., Girolami F., Nistri S., Rossi A., Rega L., Garbini F., Grifoni C., Cecchi F., Yacoub M.H. (2009). The Many Faces of Hypertrophic Cardiomyopathy: From Developmental Biology to Clinical Practice. J. Cardiovasc. Transl. Res..

[26] Maron B.J., Bonow R.O., Cannon R.O., Leon M.B., Epstein S.E. (1987). Hypertrophic cardiomyopathy: Interrelation of clinical manifestations, pathophysiology, and therapy. N. Engl. J. Med..

[27] Cannon R.O., Rosing D.R., Maron B.J., Leon M.B., Bonow R.O., Watson R.M., Epstein S.E. (1985). Myocardial ischemia in patients with hypertrophic cardiomyopathy: Contribution of inadequate vasodilator reserve and elevated ventricular filling pressures. Circulation.

[28] Raphael C.E., Cooper R., Parker K.H., Collinson J., Vassiliou V., Pennell D.J., De Silva R., Hsu L.Y., Greve A.M., Nijjer S. (2016). Mechanisms of Myocardial Ischemia in Hypertrophic Cardiomyopathy. J. Am. Coll. Cardiol..

[29] Olivotto I., Cecchi F., Gistri R., Lorenzoni R., Chiriatti G., Girolami F., Torricelli F., Camici P.G. (2006). Relevance of Coronary Microvascular Flow Impairment to Long-Term Remodeling and Systolic Dysfunction in Hypertrophic Cardiomyopathy. J. Am. Coll. Cardiol..

[30] Cecchi F., Sgalambro A., Baldi M., Sotgia B., Antoniucci D., Camici P.G., Sciagrà R., Olivotto I. (2009). Microvascular Dysfunction, Myocardial Ischemia, and Progression to Heart Failure in Patients with Hypertrophic Cardiomyopathy. J. Cardiovasc. Transl. Res..

[31] Maurizi N., Michels M., Rowin E.J., Semsarian C., Girolami F., Tomberli B., Cecchi F., Maron M.S., Olivotto I., Maron B.J. (2019). Clinical Course and Significance of Hypertrophic Cardiomyopathy Without Left Ventricular Hypertrophy. Circulation.

[32] Christiaans I., Birnie E., Bonsel G.J. (2011). Manifest disease, risk factors for sudden cardiac death, and cardiac events in a large nationwide cohort of predictively tested hypertrophic cardiomyopathy mutation carriers: Determining the best cardiological screening strategy. Eur. Heart J..

[33] Gray B., Ingles J., Semsarian C. (2011). Natural history of genotype positive-phenotype negative patients with hypertrophic cardiomyopathy. Int. J. Cardiol..

[34] Maron B.J., Spirito P., Wesley Y., Arce J. (1986). Development and progression of left ventricular hypertrophy in children with hypertrophic cardiomyopathy. N. Engl. J. Med..

[35] Spirito P., Maron B.J. (1987). Absence of progression of left ventricular hypertrophy in adult patients with hypertrophic cardiomyopathy. J. Am. Coll. Cardiol..

[36] Domenicucci S., Lazzeroni E., Roelandt J., Cate F.J.T., Vletter W.B., Arntzenius A.C., Das S.K. (1985). Progression of hypertrophic cardiomyopathy. A cross sectional echocardiographic study. Heart.

[37] Doolan G., Nguyen L., Chung J., Ingles J., Semsarian C. (2004). Progression of left ventricular hypertrophy and the angiotensin-converting enzyme gene polymorphism in hypertrophic cardiomyopathy. Int. J. Cardiol..

[38] Semsarian C., French J., Trent R.J., Richmond D.R., Jeremy R.W. (1997). The natural history of left ventricular wall thickening in hypertrophic cardiomyopathy. Aust. N. Z. J. Med..

[39] Canepa M., Fumagalli C., Tini G., Vincent-Tompkins J., Day S.M., Ashley E.A., Mazzarotto F., Ware J.S., Michels M., Jacoby D. (2020). Temporal Trend of Age at Diagnosis in Hypertrophic Cardiomyopathy: An Analysis of the International Sarcomeric Human Cardiomyopathy Registry. Circ. Heart Fail..

[40] Spirito P., Maron B.J. (1988). Relation between extent of left ventricular hypertrophy and age in hypertrophic cardiomyopathy. J. Am. Coll. Cardiol..

[41] Louie E.K., Maron B.J. (1986). Hypertrophic cardiomyopathy with extreme increase in left ventricular wall thickness: Functional and morphologic features and clinical significance. J. Am. Coll. Cardiol..

[42] Thaman R., Gimeno J.R., Reith S., Esteban MT T., Limongelli G., Murphy R.T., Mist B., McKenna W.J., Elliott P.M. (2004). Progressive left ventricular remodeling in patients with hypertrophic cardiomyopathy and severe left ventricular hypertrophy. J. Am. Coll. Cardiol..

[43] Spirito P., Maron B.J., Bonow R.O., Epstein S.E. (1987). Occurrence and significance of progressive left ventricular wall thinning and relative cavity dilatation in hypertrophic cardiomyopathy. Am. J. Cardiol..

[44] Olivotto I., Maron B.J., Appelbaum E., Harrigan C.J., Salton C., Gibson C.M., Udelson J.E., O’Donnell C., Lesser J.R., Manning W.J. (2010). Spectrum and Clinical Significance of Systolic Function and Myocardial Fibrosis Assessed by Cardiovascular Magnetic Resonance in Hypertrophic Cardiomyopathy. Am. J. Cardiol..

[45] Melacini P., Basso C., Angelini A., Calore C., Bobbo F., Tokajuk B., Bellini N., Smaniotto G., Zucchetto M., Iliceto S. (2010). Clinicopathological profiles of progressive heart failure in hypertrophic cardiomyopathy. Eur. Heart J..

[46] Ciró E., Maron B.J., Bonow R.O., Cannon R.O., Epstein S.E. (1984). Relation between marked changes in left ventricular outflow tract gradient and disease progression in hypertrophic cardiomyopathy. Am. J. Cardiol..

[47] Maron M.S., Olivotto I., Maron B.J., Prasad S.K., Cecchi F., Udelson J.E., Camici P.G. (2009). The Case for Myocardial Ischemia in Hypertrophic Cardiomyopathy. J. Am. Coll. Cardiol..

[48] Musumeci M.B., Russo D., Limite L.R., Canepa M., Tini G., Casenghi M., Francia P., Adduci C., Pagannone E., Magri D. (2018). Long-Term Left Ventricular Remodeling of Patients with Hypertrophic Cardiomyopathy. Am. J. Cardiol..

[49] Harris K.M., Spirito P., Maron M.S., Zenovich A.G., Formisano F., Lesser J.R., Mackey-Bojack S., Manning W.J., Udelson J.E., Maron B.J. (2006). Prevalence, Clinical Profile, and Significance of Left Ventricular Remodeling in the End-Stage Phase of Hypertrophic Cardiomyopathy. Circulation.

[50] Biagini E., Coccolo F., Ferlito M., Perugini E., Rocchi G., Bacchi-Reggiani L., Lofiego C., Boriani G., Prandstraller D., Picchio F.M. (2005). Dilated-hypokinetic evolution of hypertrophic cardiomyopathy: Prevalence, incidence, risk factors, and prognostic implications in pediatric and adult patients. J. Am. Coll. Cardiol..

[51] Kubo T., Gimeno J.R., Bahl A., Steffensen U., Steffensen M., Osman E., Thaman R., Mogensen J., Elliot P.M., Doi Y. (2007). Prevalence, clinical significance, and genetic basis of hypertrophic cardiomyopathy with restrictive phenotype. J. Am. Coll. Cardiol..

[52] Rowin E.J., Maron B.J., Haas T.S., Garberich R.F., Wang W., Link M.S., Maron M.S. (2017). Hypertrophi cardiomyopathy with left ventricular apical aneurysm. J. Am. Coll. Cardiol..

[53] Maron M.S., Rowin E.J., Maron B.J. (2020). Hypertrophic cardiomyopathy with left ventricular apical aneurysm: The newest high-risk phenotype. Eur. Heart J. Cardiovasc. Imaging.

[54] Kubo T., Kitaoka H., Okawa M., Yamanaka S., Hirota T., Baba Y., Hayato K., Yamasaki N., Matsumura Y., Yasuda N. (2011). Combined Measurements of Cardiac Troponin I and Brain Natriuretic Peptide Are Useful for Predicting Adverse Outcomes in Hypertrophic Cardiomyopathy. Circ. J..

[55] Pennacchini E., Musumeci M.B., Conte M.R., Stöllberger C., Formisano F., Bongioanni S., Francia P., Volpe M., Autore C. (2015). Electrocardiographic evolution in patients with hypertrophic cardiomyopathy who develop a left ventricular apical aneurysm. J. Electrocardiol..

[56] Habib M., Adler A., Fardfini K., Hoss S., Hanneman K., Rowin E.J., Maron M.S., Maron B.J., Rakowski H., Chan R.H. (2020). Progression of myocardial fibrosis in HCM. JACC Cardiovasc. Imaging.

[57] Chan R.H., Maron B.J., Olivotto I., Pencina M.J., Assenza G.E., Haas T., Lesser J.R., Gruner C., Crean A.M., Rakowski H. (2014). Prognostic Value of Quantitative Contrast-Enhanced Cardiovascular Magnetic Resonance for the Evaluation of Sudden Death Risk in Patients With Hypertrophic Cardiomyopathy. Circulation.

[58] Rowin E.J., Maron B.J., Carrick R.T., Patel P.P., Koethe B., Wells S., Maron M.S. (2020). Outcomes in Patients with Hypertrophic Cardiomyopathy and Left Ventricular Systolic Dysfunction. J. Am. Coll. Cardiol..

[59] Musumeci M.B., Mastromarino V., Casenghi M., Tini G., Francia P., Maruotti A., Romaniello A., Magri D., Lillo R., Adduci C. (2017). Pulmonary hypertension and clinical correlates in hypertrophic cardiomyopathy. Int. J. Cardiol..

